# CHATO NATIONAL TRIAL: USING OPEN-ENDED RESPONSE TO DEVELOP VIDEO TUTORIALS TO IMPROVE NURSING HOME IMPLEMENTATION

**DOI:** 10.1093/geroni/igae098.3355

**Published:** 2024-12-31

**Authors:** Maria Jaros, Carissa Coleman, Frances Yang, Clarissa Shaw, Maria Hein, Yelena Perkhounkova, Kristine Williams

**Affiliations:** University of Kansas Medical Center, Kansas City, Kansas, United States; University of Kansas Medical Center, Kansas City, Kansas, United States; University of Kansas Medical Center, Kansas City, Kansas, United States; University of Iowa, Iowa City, Iowa, United States; University of Iowa, Iowa City, Iowa, United States; University of Iowa, Iowa City, Iowa, United States; University of Kansas Medical Center, Kansas City, Kansas, United States

## Abstract

The Changing Talk: Online (CHATO) intervention was developed to increase staff access to person-centered dementia care communication education in a national nursing home sample. A qualitative review of the post-evaluation survey responses (N=1605) from participants in the first year of the study was performed to determine issues associated with the modules to improve implementation across nursing homes. Three open-ended questions addressed technical difficulties and what participants liked most and least about the program. Technical difficulties were reported by 21.1% of participants, including internet connection (5%), video difficulties (3.8%), non-specific technical difficulties (3.7%), website interface (3.1%), registration (1.8%), and navigating the modules (1.1%). Sixty-nine percent of participants reported what they liked the best about the course. Participants indicated real-world application (22.4%) was liked best, followed by the videos in the modules (18.8%), and the presentation of the material (5.9%). When asked what participants liked least about the course, 54.3% of participants provided input. The length of the three-hour course (21.4%) was liked least, followed by the presentation of the material (7.7%), and the interactive components of the modules (6.5%). In response to these findings, video tutorials were created addressing components most reported in the post-evaluation which included outlining navigation features within the CHATO training, registering for the course, and navigating to the modules. Tracking new users accessing the video tutorials via the CHATO website as well as annual coding will provide ongoing monitoring of implementation success and will provide input for re-design of the modules in future research.

